# Dental Surgery

**Published:** 1856-01

**Authors:** 


### DENTAL SURGERY.

It gives us much pleasure to be able to testify in favor of this department in our city. We see no good reason why persons requiring this kind of professional service should go elsewhere. We have seen as fine artificial teeth made and fitted here, as we have observed elsewhere. We can now point to some setts made in this city, which we believe cannot be excelled by any Dental Surgeon.

Dr. Rickey, some years a resident here, has his neat and tasteful office on Blondeau street, where he is kept very much engaged. He is well known in this city as a good dentist. Dr. Hicks has erected a fine brick office on Second street, and his dental rooms are not excelled for their taste, or the number or quality of appliances for operating, by any other in the west. Then there is our young friend, Dr. Barber, on Third, between Main and Johnson, who has shown himself highly skillful in his profession, by the construction of some setts of teeth, which, for neatness and adaptation, are not excelled. The Doctor is a genius in artistical skill. All are well supplied with all the needed appliances for operating, all have comfortable, or rather, elegant rooms, in which their patients can be made as comfortable as they desire, and all are gentlemen. This we have believed our duty to say, without solicitation from any quarter.

